# Supplementary material for: GJB2 and GJB6 Mutations in Hereditary Recessive Non-Syndromic Hearing Impairment in Cameroon
Source: Genes (Basel). 2019 Oct 25;10(11):844. doi: 10.3390/genes10110844 (PMC6895965; doi:10.3390/genes10110844)
Supplement: Supplementary file 1 [file genes-10-00844-s001.pdf]

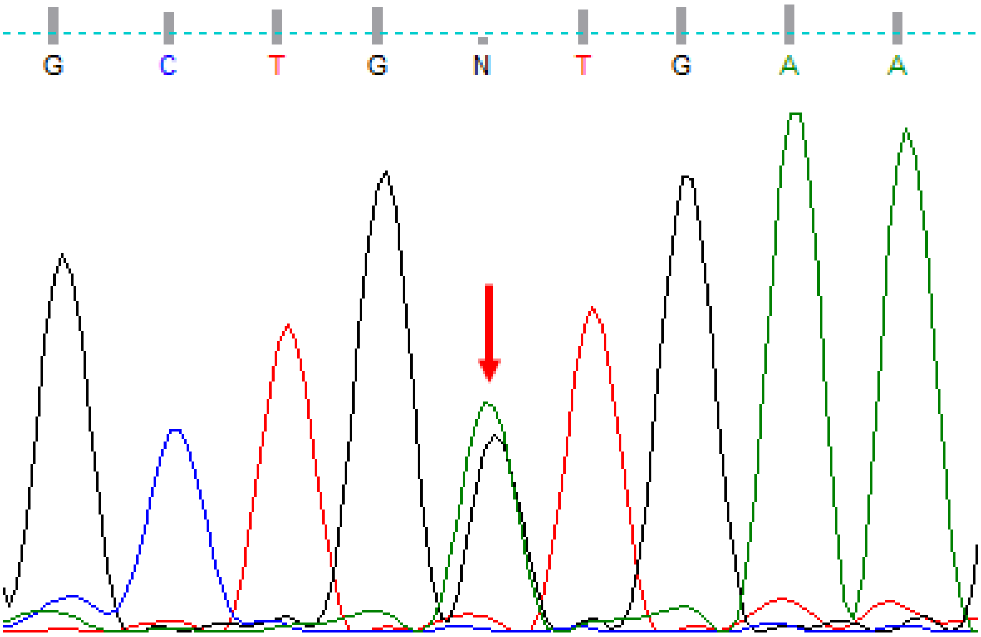

Figure S1. *GJB2* variant of uncertain significance, c.499G>A (p.V167M), present in a family in the heterozygous form.

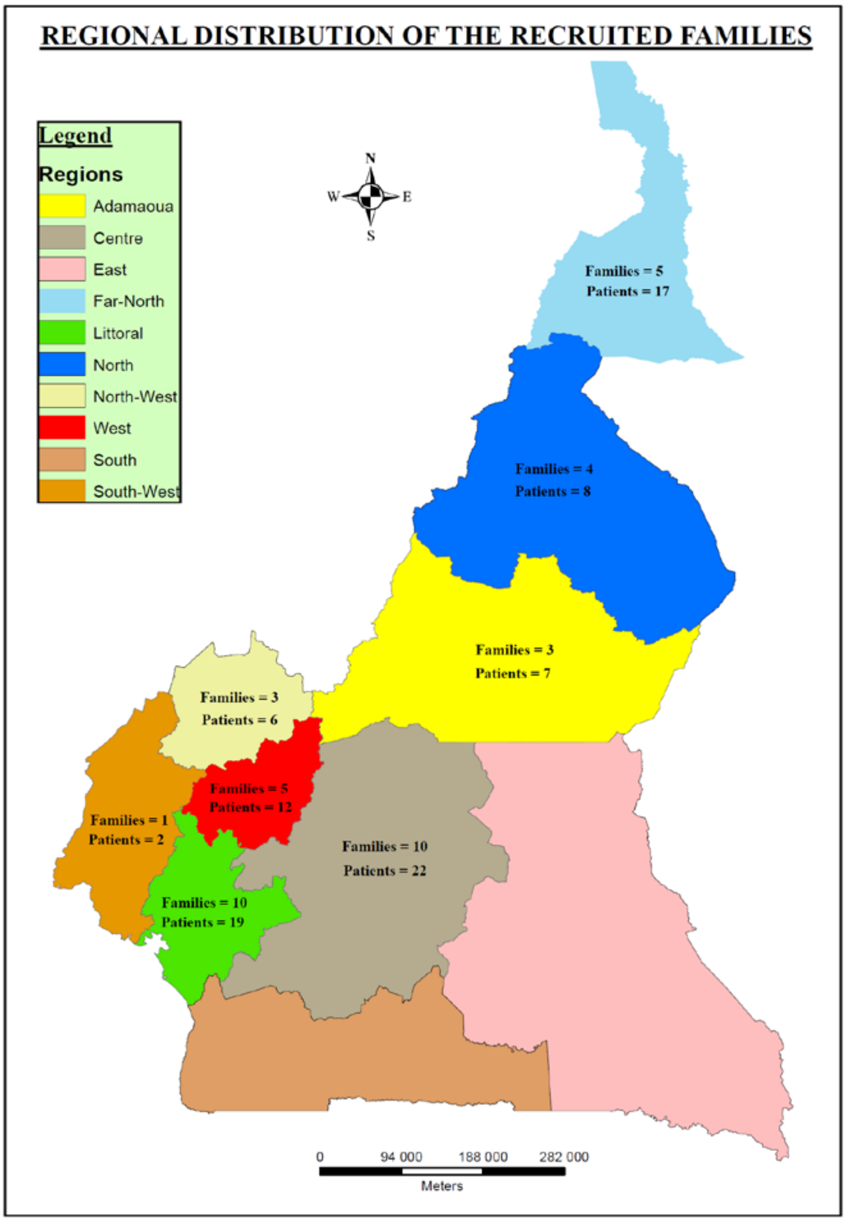

Figure S2. Regional distribution of Families.
